# Bioinformatics Analysis of Long Non-coding RNA and Related Diseases: An Overview

**DOI:** 10.3389/fgene.2021.813873

**Published:** 2021-12-08

**Authors:** Yuxin Gong, Wen Zhu, Meili Sun, Lei Shi

**Affiliations:** ^1^ School of Mathematics and Statistics, Hainan Normal University, Haikou, China; ^2^ Yangtze Delta Region Institute (Quzhou), University of Electronic Science and Technology of China, Quzhou, China; ^3^ Key Laboratory of Computational Science and Application of Hainan Province, Haikou, China; ^4^ Key Laboratory of Data Science and Smart Education, Hainan Normal University, Ministry of Education, Haikou, China; ^5^ Beidahuang Industry Group General Hospital, Harbin, China; ^6^ Department of Spine Surgery, Changzheng Hospital, Naval Medical University, Shanghai, China

**Keywords:** lncRNA, database, mechanism of action, recognition methods, disease

## Abstract

Long non-coding RNAs (lncRNAs) are usually located in the nucleus and cytoplasm of cells. The transcripts of lncRNAs are >200 nucleotides in length and do not encode proteins. Compared with small RNAs, lncRNAs have longer sequences, more complex spatial structures, and more diverse and complex mechanisms involved in the regulation of gene expression. LncRNAs are widely involved in the biological processes of cells, and in the occurrence and development of many human diseases. Many studies have shown that lncRNAs can induce the occurrence of diseases, and some lncRNAs undergo specific changes in tumor cells. Research into the roles of lncRNAs has covered the diagnosis of, for example, cardiovascular, cerebrovascular, and central nervous system diseases. The bioinformatics of lncRNAs has gradually become a research hotspot and has led to the discovery of a large number of lncRNAs and associated biological functions, and lncRNA databases and recognition models have been developed. In this review, the research progress of lncRNAs is discussed, and lncRNA-related databases and the mechanisms and modes of action of lncRNAs are described. In addition, disease-related lncRNA methods and the relationships between lncRNAs and human lung adenocarcinoma, rectal cancer, colon cancer, heart disease, and diabetes are discussed. Finally, the significance and existing problems of lncRNA research are considered.

## Introduction

**FIGURE 1:**
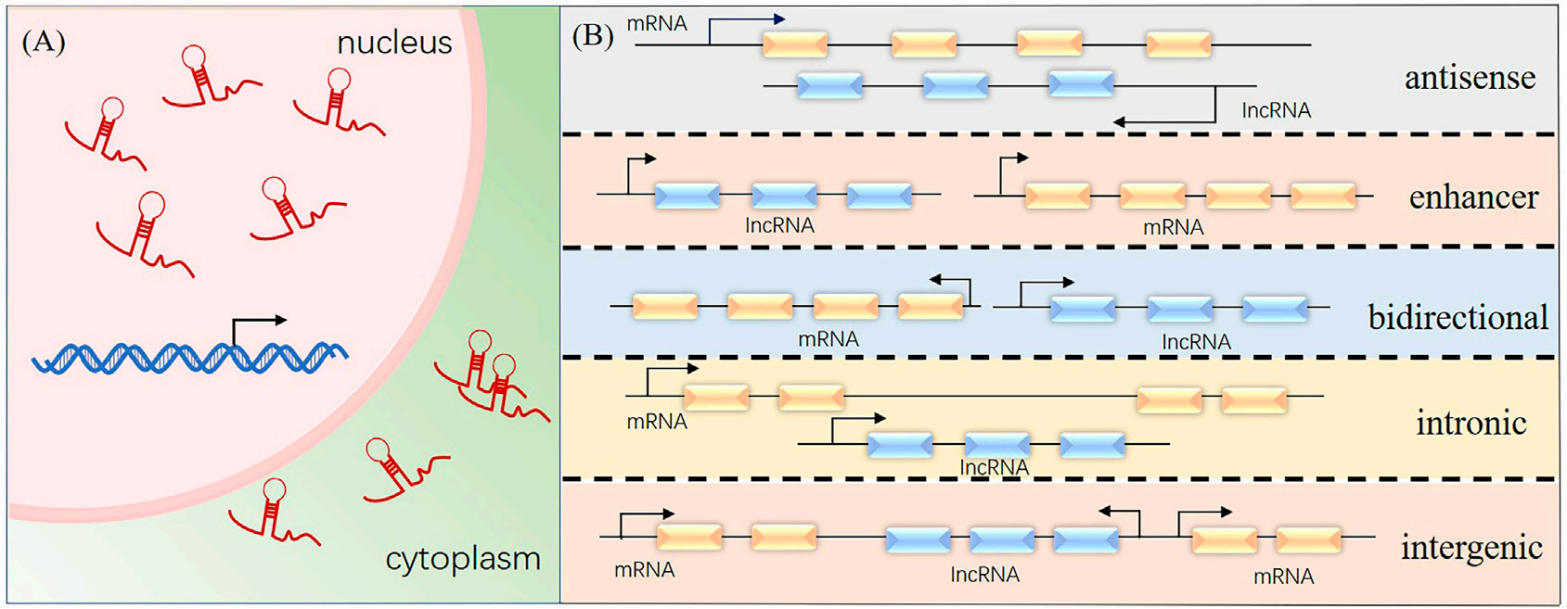
Cellular localization and classification of long non-coding RNAs (lncRNA). **(A)** Cellular distribution of lncRNAs. **(B)** Positions of lncRNAs in the genome relative to the target protein-coding gene.

A long transcription product was discovered and identified by Okazaki in 2002 (Okazaki et al., 2003) when sequencing a mouse cDNA library. The transcript was called long non-coding RNA (lncRNA). LncRNAs are >200 nucleotides long and similar in structure to messenger RNAs, but they lack an open reading frame. Mainly distributed in the nucleus and cytoplasm of cells, lncRNAs are the transcription products of RNA polymerase II. LncRNAs have been classified based on the lengths of the coded transcripts as lncRNA, long-intergenic non-coding RNA, very long-intergenic non-coding RNA, macroRNA, and promoter-associated long RNA. They have also been classified according to the position of the lncRNA in the genome relative to the target protein-coding gene as 1) antisense lncRNA, which is partially or completely complementary to the transcription product on the opposite strand; 2) enhancer lncRNA, which is produced from the enhancer region of a protein coding gene; 3) bidirectional lncRNA, which shares the same promoter with protein-coding genes, but the transcription direction is opposite; 4) intronic lncRNA, which is produced by introns of genes; and 5) large intergenic non-coding RNA, which is independently transcribed from sequences located between protein-coding genes (Figure 1).

Because non-coding RNAs (ncRNAs) do not encode proteins, they were thought to have no biological function and were regarded as “transcriptional noise” for a long time after they were discovered. Although, research on short ncRNAs, including microRNAs, short interfering RNAs, small nucleolar RNAs, and Piwi-interacting RNAs, has flourished, lncRNAs have been neglected because of their long sequences and limitations of the research methods (Liu, 2021).

Initially, lncRNAs were considered to be the noise of genome transcription and a by-product of polymerase II transcription. However, after lncRNAs were found to have conserved secondary structures, spliced forms, and subcellular localizations, it was realized that they may be functional. Indeed, it is now recognized that lncRNAs have functions that are essential for many biological processes, including epigenetic regulation, cell signal transduction, immune response, and cell proliferation and differentiation (Heo et al., 2013; Zhao et al., 2021; Yang et al., 2020; Hu et al., 2021; Hu et al., 20216222), and the abnormal expression of lncRNAs can result in a variety of complex diseases. Moreover, some lncRNAs can act as precursors of some functional short ncRNAs to indirectly participate in the regulation of target genes. LncRNAs regulate gene expression to exert these functions, including *cis*-regulation and *trans*-regulation.

According to data from the Encyclopedia of DNA Elements (ENCODE) project (2015), approximately 15,941 lncRNA loci have been identified in the human genome (Jalali et al., 2016). The discovery and research of a large number of lncRNAs have greatly promoted the development of RNA biology research. Analysis of these lncRNAs showed that they had important regulatory functions at the epigenetic, transcription, and post-transcriptional levels (Mercer et al., 2009; Hu et al., 2020; Shen et al., 2021). Until now, research on the relationship between lncRNA and disease has been focused mainly on tumors; however, a small number of differentially expressed lncRNAs associated with obesity, diabetes, hypertension, and other diseases have been found, but the mechanism of action is still unclear. LncRNA research suffers from problems such as few available resources, relatively independent research results, and lack of systematicness (Losko et al., 2016). In-depth analysis of disease-related lncRNAs will help in finding disease biomarkers and provide insights into the diagnosis, treatment, prognosis, and prevention of diseases (Tang et al., 2018; Sun et al., 2021). In this review, bioinformatics approaches for the study of lncRNAs and diseases are summarized, including mainly databases related to lncRNAs, the mechanisms and modes of lncRNA action, methods for identifying disease-related lncRNAs, and the relationships between lncRNAs and human metabolic syndrome, cancer, leukemia, heart disease, and neurodegenerative diseases.

## Databases Related to LncRNAs

A large number of biological datasets related to lncRNA have been generated and many lncRNA-oriented databases have been built to store, manage, and integrate comprehensive lncRNA functional information, lncRNA structure and genome mutations, lncRNA expression analysis data, and lncRNA–disease associations. Here, the commonly used lncRNA databases are briefly described.

1) ChIPBase (Zhou et al., 2016) provides comprehensive identification and annotation of lncRNA expression profiles and transcriptional regulation data. The lncRNA expression profiles obtained by RNA sequencing and transcription factor binding sites identified by ChIP-Seq (chromatin immunoprecipitation followed by sequencing) are included in this database (http://rna.sysu.edu.cn/chipbase3/index.php).
2) lncRNAdb (Quek et al., 2015) contains comprehensive annotations of lncRNAs with biological functions, including gene expression, functional evidence, disease-related lncRNAs, pathogen-induced or derived lncRNAs, and sequence information (http://www.lncrnadb.org/).
3) NRED (Dinger et al., 2009) provides the expression information of thousands of lncRNAs of humans and mice from microarray and *in situ* hybridization data, as well as auxiliary information such as secondary structure evidence, antisense relationships, evolutionary conservation, and genome-related text links (http://jsm-research.imb.uq.edu.au/nred/).
4) NONCODE (Fang et al., 2018) provides comprehensive lncRNA annotations, including expression information and functions predicted by the ncFANs software. NONCODE is widely used for ncRNA research (http://www.noncode.org).
5) LncRNADisease (Bao et al., 2019) contains annotations of disease-related lncRNAs reported in the literature. For the lncRNAs, basic information such as name, chromosome location, species, transcript number, and sequence is provided. For the related diseases, name, literature, and other information are given (http://www.rnanut.net/lncrnadisease/).
6) lncRNASNP2 (Miao et al., 2017) provides resources of single nucleotide polymorphisms (SNPs) in lncRNAs of humans and mice. The database has the following browse and search functions: the influence of SNPs in lncRNAs on their own genes, the binding of microRNAs (miRNAs) in lncRNAs and the influence of SNPs on binding, the mutation and expression of lncRNAs in The Cancer Genome Atlas, variations in lncRNAs in the COSMIC database (large databases of cancer-related somatic mutation sites), and the effect of mutations on lncRNAs (http://bioinfo.life.hust.edu.cn/lncRNASNP#!/).
7) StarBase v2.0 (Li et al., 2013) can search for lncRNA based on miRNA-lncRNA interaction, as well as competitive endogenous RNA regulatory molecules and binding proteins related to the specified lncRNAs (http://starbase.sysu.edu.cn/index.php).

## Mechanisms and Modes of Action of LncRNAs Headings

Compared with small RNAs, lncRNAs are longer, have more complex spatial structure, and more diverse and complex mechanisms for expression regulation. LncRNAs are involved in the regulation of development, differentiation, and metabolism. LncRNAs can regulate gene expression at the epigenetic (Mercer and Mattick, 2013), transcriptional (Bonasio and Shiekhattar, 2014), and post-transcriptional (Yoon et al., 2013) levels. They also participate in important regulatory processes such as X chromosome silencing, genome imprinting, chromatin modification, transcription activation and inhibition, and nuclear transport. LncRNAs are also closely related with the occurrence, development, and prevention of human diseases. The mechanisms of lncRNA action discovered so far include X chromosome inactivation and genome imprinting, chromatin modification, cell cycle regulation and apoptosis, mRNA decay, and protein translation regulation (Zou et al., 2019).

**FIGURE 2:**
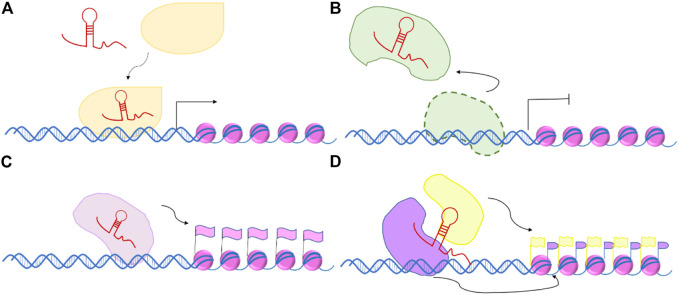
Modes of action long non-coding RNAs. **(A)** Signal, **(B)** Decoy, **(C)** Guide, and **(D)** Scaffold.

The modes of lncRNA action are signal, decoy, guide, and scaffold (Wang and Chang, 2011) as shown in Figure 2. The molecular functions of lncRNAs can be explained based on the mode of action. In the signal mode, lncRNAs participate in gene imprinting processes. For example, the lncRNAs Kcnq1ot1 and Xist both function in the signal mode. The mode of action of Kcnq1ot1 and Xist are similar. Kcnq1ot1 binds to the chromosome and inhibits the expression of Kcnq1 by recruiting H3K9- and H3K27-specific histone methyltransferases and polycomb repressive complex 2 (PRC2) complexes. This function is hereditary (Pandey et al., 2008). In cells with two X chromosomes, one of the X chromosomes is suppressed. Xist regulates the process by which the X chromosome is selectively suppressed and maintains this phenotype to the next generation (Plath et al., 2002).

In the decoy mode, lncRNAs bind to proteins that have transcriptional regulatory functions (e.g., transcription factors and chromosome folding proteins), thereby regulating the transcriptional activation and inhibition of related genes by controlling the activity of molecules and signal pathways. LncRNA Gas5 functions in the decoy mode. It binds to the DNA-binding domain of the glucocorticoid receptor through the RNA motif to inhibit the physiological function of the receptor (Kino et al., 2010). LncRNA can also be used as a molecular decoy for miRNAs and splicing factors, inhibiting their functions. MiRNAs can promote the formation of protein complexes, hence playing an important role in gene regulation. LncRNA PTENP1 can inhibit human tumors. In the decoy mode of action, PTENP1 binds to a group of miRNAs that act on the PTEN 3′ untranslated region, thereby regulating the expression of PTEN (Poliseno et al., 2010).

In the guide mode, lncRNAs bind to proteins to guide the protein complex to a specific DNA sequence, thereby regulating the transcription of downstream molecules. The guiding can be either cis or trans. One *cis*-regulatory mechanism of lncRNA involves the inactive center of the X chromosome, which controls the silence of the maternal X chromosome. A *trans*-regulatory mechanism included reducing the expression of tumor-associated lncRNA HOTAIR and decreasing cell invasiveness.

In the scaffold mode, lncRNAs simultaneously bind multiple related transcription factors to provide a platform for interaction. For example, lncRNA HOTAIR splices and bridges between the PRC2 and LSD1 complexes. The 5′ end of HOTAIR combines with the PRC2 complex (acting on H3 and H27 to methylate them) to promote gene expression (Rinn et al., 2007), whereas the 3′ end of HOTAIR combines with LSD1 (acting on H3K4 to demethylate it) to antagonize gene expression activation (Tsai et al., 2010), thereby inducing the interaction between the PRC2 and LSD1 complexes.

## Methods to Identify Disease-Related LncRNAs

LncRNAs by far outnumber protein-coding genes, and, unlike protein-coding genes, lncRNAs are usually not conserved in sequence fragments and secondary structures, which make lncRNA functional prediction difficult. Two main approaches have been used to identify disease-related lncRNAs, methods based on biological experiments and methods based on computational predictions. The former generally produces more reliable results, but the cost is high and the efficiency is low; the latter uses multi-source biological data, such as disease-related genomes, transcriptomes, and proteomes, to predict disease-related lncRNAs. Bioinformatics methods have been applied to predict disease-related lncRNAs based on the analysis of multi-source biological data (Cao et al., 2021).

### Identification of Disease-Related lncRNA by Biological Experiments

Large-scale biological experimental research is often limited by ethical factors that govern the collection of experimental samples. However, the influence of interference factors on the experimental results can often be largely controlled, and therefore the results are likely to be objective and highly reliable. Two examples of lncRNA research using biological experiments are briefly described.

Nakagawa et al. (Nakagawa et al., 2014) found that approximately 50% of lncRNA Neat1 knockout mice with abnormal ovulation did not become pregnant, and this outcome seemed to be random. Subsequently, corpus luteum dysfunction and the accompanying low progesterone were found to contribute most to the decline in fertility. Unlike the weak expression of Neat1 observed in most adult tissues, in the infertile Neat1 knockout mice, Neat1 was highly expressed in the corpus luteum and the formation of the corpus luteum was severely impaired. These results indicated that Neat1 may be closely related to the formation of the corpus luteum and some forms of infertility in humans.

Zhang et al. (Zhang et al., 2018) screened and identified a new type of lncRNA, HOXC-AS3, using publicly available gastric cancer expression profile data and integrated bioinformatics analysis. They found that the expression of HOXC-AS3 was highly up-regulated in gastric cancer tissues and was related to clinicopathological factors such as histological grade, depth of tumor invasion, lymph node metastasis, and poor prognosis. They performed chromatin immunoprecipitation assays to explore the mechanisms involved in the high HOXC-AS3 expression, and found that HOXC-AS3 was partially activated by H3K4me3 and H3K27ac in cells and tissues. Overexpression and knockout of HOXC-AS3 were used to detect cell apoptosis and proliferation. They found that overexpression of HOXC-AS3 promoted the proliferation of cancer cells, and knockout of HOXC-AS3 induced apoptosis of cancer cells. To further explore the mechanism of action of HOXC-AS3, the transcription factor YBX1 was selected, and three independent RNA pull-down mass spectrometry analyses were performed. The results showed that HOXC-AS3 interacted with YBX1. This result combined with the results of the immunoprecipitation assays, confirmed that YBX1 was involved in HOXC-AS3-mediated gene transcription regulation in gastric cancer.

Clearly, traditional biological experiments are not only time-consuming but also expensive. Computational models are less time-consuming and less expensive, therefore they have attracted more and more attention as a solution that can predict lncRNA functions on a large scale. Models can be used to predict the possible functions of lncRNAs according to related priorities, and the predictions can be verified experimentally. This process effectively promotes the functional recognition of lncRNAs.

### Identification of Disease-Related LncRNAs by Computational Prediction

Because biological experiments are costly and time-consuming, the use of bioinformatics calculations to predict disease-related lncRNAs has become the mainstream. In recent years, many lncRNA-disease association prediction (LDAP) models have been proposed, including models based on biological networks, models that do not rely on known lncRNA–disease associations, and models based on machine learning algorithms. The three types of LDAP models are briefly described.

#### LDAP Models Based on Biological Networks

These models integrate biological networks such as the disease similarity network, lncRNA similarity network, and lncRNA–disease association network to construct an LDAP model. In 2014, Sun et al. (Sun et al., 2014) proposed an LDAP model based on the functionally similar network of lncRNAs and random walks with restart. In 2015, Chen et al. (Weng et al., 2015) developed the KATZLDA prediction model by fusing lncRNA–disease association, lncRNA expression profile, lncRNA functional similarity, and disease semantic similarity data. In 2017, Yu et al. (Yu et al., 2017) developed the BRWLDA prediction model based on double random walks, and Gu et al. (Gu et al., 2017) developed a GRWLDA prediction model based on global network random walk. In 2018, Ping et al. (Ping et al., 2019) proposed a prediction model based on the known lncRNA–disease association network to infer potential lncRNA–disease associations. In 2019, Fan et al. (Fan et al., 2019) proposed an LDAP model based on multiple heterogeneous information networks and random walks with restart. Xie et al. (Xie et al., 2019) proposed an SFK-LDA prediction model based on similarity nuclear fusion. In 2020, Zhou et al. (Zhou et al., 2021) built a heterogeneous network by integrating various associations between diseases and miRNAs, lncRNAs, proteins, and drugs, and trained a LDAP model with the rotating forest classifier, and Zhang et al. (Zhang et al., 2020) proposed an LDAP model based on network feature similarity and gradient boosting. In 2021, Liu et al. (Liu et al., 2021) proposed an LDAP model based on the weighted graph regularized collaborative matrix factorization.

#### LDAP Models That do not Rely on Known LncRNA–Disease Associations

In these models, the expression and regulatory relationship between disease-related genes or miRNAs and lncRNAs are used to predict potential lncRNA–disease associations. In 2014, Liu et al. (Zhao et al., 2015) developed the first LDAP model that did not rely on known lncRNA–disease associations by integrating lncRNA expression profiles, gene expression profiles, and disease-related gene data. In 2015, Chen et al. (Chen, 2015) developed an LDAP model HGLDA based on hypergeometric distribution by integrating miRNA–disease associations and ncRNA–miRNA interactions. In 2016, Cheng et al. (Cheng et al., 2016) proposed the IntNetLncSim computing framework, which inferred the functional similarity of lncRNAs and predicted new lncRNA–disease associations by modeling the information flow in an integrated network that contained lncRNA transcription and post-transcription information. In 2017, Wang et al. (Wang et al., 2017) mapped lncRNAs to their functional genomic context based on the theory of competing endogenous RNAs to predict new lncRNA–disease associations. Fu et al. (Fu et al., 2018) proposed a matrix decomposition-based LDAP model MFLDA, which decomposed the data matrix of heterogeneous data sources into low-rank matrices through matrix decomposition to explore and use their internal and shared structure. In 2018, Ding et al. (Ding et al., 2018) proposed an LDAP model based on a lncRNA–disease–gene network that integrated gene–disease and lncRNA–disease associations. In 2020, Xiao et al. (Xiao et al., 2020) proposed an LDAP model that used both direct and indirect features of lncRNA–disease relationship pairs, and Tang et al. proposed a hierarchical extended LDAP model based on a Boolean matrix (Tang et al., 2020).

#### LDAP Models Based on Machine Learning Algorithms

These models integrate biological data and use various machine learning algorithms to predict disease-related lncRNAs. In 2013, Chen et al. (Chen and Yan, 2013) developed a semi-supervised learning framework LRLSLDA based on Laplace regularization least squares by integrating lncRNA expression profiles and known lncRNA–disease associations. In 2015, Liu et al. (Liu et al., 2015) developed an LDAP model based on the naive Bayes classifier to identify lncRNAs related to cancer by integrating genome, regulatory factors, and transcriptome data. In 2017, Lan et al. (Lan et al., 2017) proposed an LDAP model based on support vector machines. In 2018, Yu et al. (Yu et al., 2018) proposed the NBCLDA model based on the naive Bayes classifier. In 2019, Guo et al. (Guo et al., 2019) proposed two LDAP models, one based on rotating forest and neural network and another based on a random forest classifier. Sheng et al. (Sheng et al., 2021) proposed a series of LDAP models based on convolutional neural networks, including CNNLDA, as well as an attention multi-level representation coding model based on convolution and variance autoencoders. In 2020, Zeng et al. (Zeng et al., 2020) proposed SDLDA, an LDAP model based on singular value decomposition and deep learning. Fan et al. (Fan et al., 2020) proposed IDSSIM, a calculation model of lncRNA functional similarity based on improved disease semantic similarity. Tan et al. (Tan et al., 2020) proposed a multi-view consensus graph learning model to predict lncRNA–disease association. Wei et al. (Wei et al., 2021) proposed a convolutional neural network model fused with multiple biological characteristics to predict lncRNA–disease association.

## LncRNAs and Related Diseases

LncRNAs induce the occurrence of disease by regulating disease-related protein coding genes, thus leading to improper expression of lncRNAs or altering the chromatin that contain disease-related gene polymorphisms and non-coding regions. Therefore, the expression of lncRNAs is important in the diagnosis, occurrence, development, and treatment of diseases. In recent years, the associations of lncRNAs with cancer, leukemia, cardiovascular and cerebrovascular diseases, diabetes, and other diseases have been a focus of study. Future clinical applications of disease-related lncRNAs are very likely.

The roles of lncRNAs in cancer: Li et al. (Li et al., 2016) found that up-regulation of lncRNA MALAT1 was related to tumor size and lymph node metastasis, and to the shorter overall survival of patients with lung adenocarcinoma. *In vivo* and *in vitro* experiments showed that MALAT1 promoted epithelial–mesenchymal transition and metastasis of lung adenocarcinoma cells. Numerous lncRNAs have been found to encode small proteins or micropeptides, some of which play roles in diseases. Meng et al. (Meng et al., 2020) found that lncRNA LOC90024 encodes a splicing regulatory small protein that induces the formation of Sp4 transcription factor splice variants, thereby promoting the occurrence and development of advanced rectal cancer tumors. Zhu et al. (Zhu et al., 2020) found that lncRNA LINC00266-1 encodes an RNA-binding regulatory peptide that, when highly expressed in patients with colon cancer, leads to a poor prognosis. The oncogenic peptide encoded by LINC00266-1 exerts its carcinogenic function by enhancing the recognition of N6-methyladenosine of RNA.

The roles of lncRNAs in leukemia: Garzon et al. (Garzon et al., 2014) developed a prognostic scoring system to determine if lncRNAs were associated with cytogenetically normal acute myeloid leukemia (CN-AML) clinical features and recurrent mutations in patients older than 60 years. First, 48 lncRNAs most relevant to prognosis were identified. Then, patients with CN-AML were divided into two groups, those with good prognostic scores and those with poor prognostic scores, based on the 48 lncRNAs. The prognostic scores were verified in an independent matched group of patients with CN-AML who received the same treatment. The comparative analysis showed that the lncRNA expression profile was closely related to the recurrent mutation and expression of AML, implying that some of 48 lncRNAs may have a functional role in the development of leukemia. These lncRNAs are good candidates as biomarkers for the prognosis of AML.

The roles of lncRNAs in heart disease: Han et al. (Han et al., 2014) developed a new lncRNA–chromatin mechanism to treat heart failure. A lncRNA transcript Mhrt779 from myosin heavy chain 7 loci was found to be specifically expressed in cardiomyocytes and to gradually increase with the development of embryos, especially after birth. Furthermore, the Brg1–Hdac–Parp chromatin inhibitory complex was activated by pathological stress and lncRNA Mhrt transcription was inhibited in the heart, thereby protecting the heart from hypertrophy and failure. These results show that there is a conserved lncRNA mechanism in human cardiomyopathy, and also establish a new paradigm for lncRNA–chromatin interactions.

The roles of lncRNAs in neurodegenerative diseases: Alzheimer’s disease is a progressively developing neurodegenerative disease with insidious onset that is believed to be caused by a large amount of amyloid *β*-protein (Aβ) expression, which leads to pathological changes in patients. Aβ is hydrolyzed from *β*-amyloid precursor protein, and excessive Aβ deposition can cause degenerative diseases related to neurons. BACE1 is a key enzyme in the production of Aβ. Faghihi et al. (Faghihi and Wahlestedt, 2009) found that lncRNA BACE1-AS (antisense transcript of BACE1) increased the stability of BACE1 mRNA through a mechanism that involved the formation of RNA duplexes, and this in turn facilitated the accumulation of Aβ in patients with Alzheimer’s disease.

The roles of lncRNAs in diabetes: LncRNAs in human pancreatic *β*-cells exhibit dynamic regulation during differentiation or when glucose concentrations change. Akerman et al. (Akerman et al., 2017) studied the functions of *β*-cell-specific lncRNAs and transcription factors using transcript knockdown and co-expression network analysis strategies. They found that lncRNAs and transcription factors acted synergistically to regulate the specific transcription network of *β*-cells. LncRNA PLUTO affected local three-dimensional chromatin structure and transcription of PDX1, which encodes a key *β*-cell transcription factor. PLUTO and PDX1 were both down-regulated in islets from donors with type 2 diabetes or impaired glucose tolerance. These results indicate the role of lncRNAs in *β*-cell gene regulation and diabetes.

## Challenges and Research Prospects

Compared with protein-coding sequences and small RNA molecules, lncRNA-related research is insufficient and there are many problems still to be solved. The major ones are listed here.

1) No standardized naming of lncRNAs. Until now, lncRNAs have been named according to their functions, structural characteristics, or modes of action. Therefore, it is difficult to understand their roles and functions from the name.
2) Unannotated and unbalanced lncRNA data. Compared with other ncRNA databases, the annotation information in lncRNA databases is insufficient, and disease-related lncRNAs that are included in multi-source data have problems such as serious imbalances of information.
3) Lack of lncRNA-specific technologies. Because of the diverse types and functions of lncRNAs, more effective methods are needed for systematically studying the biological functions of lncRNAs and for identifying disease-related lncRNAs. The process of combining multi-data to predict disease-related lncRNAs has problems, such as high-dimensional feature space, high noise, and redundant feature interference, that seriously affect the accuracy of the predictions.
4) Research fields need to be expanded. Current research on lncRNAs has focused mainly on tumors, nerves, and development. However, the genetic characteristics of cancer-related lncRNAs and the mechanism of action of complex diseases are still unclear. More areas of disease research related to lncRNAs need to be developed.

Despite these problems, lncRNA research has continuously advanced the understanding of lncRNAs. lncRNAs not only exert their biological functions in a variety of mechanisms in different organisms, but their dysfunction can lead to the occurrence and development of many diseases. Undoubtedly, new technologies and new methods will be developed for use in lncRNA bioinformatics research. Such developments will help to further analyze the functions and regulation mechanisms of lncRNAs, as well as the pathological mechanisms associate with the development of diseases.

## Discussion

LncRNAs are closely related to cell cycle and differentiation, aging and human diseases. Therefore, the research on their functions and mechanisms is also constantly deepening. This review summarized the following key points:1) according to the four modes of action of lncRNA, the corresponding molecular functions were described respectively. 2) The identification methods of lncRNA related to diseases were summarized into two parts: lncRNA identification research based on biological experiments and computational prediction. 3) The relationship between lncRNA and various human diseases was expounded.

Although the research technology of lncRNA is constantly developing, there are still a number of limitations:1) The low abundance of lncRNAs and lack of annotation information lead to inaccurate positioning. 2) The data set of lncRNAs in the database is not perfect. 3) The types of diseases associated with lncRNA are limited. Therefore, in the future, researchers need to continue to dig out the functional information of unknown lncRNAs and develop new lncRNA recognition models, which will help to enhance the scientific understanding of more human diseases.
